# Tristetraprolin inhibits mitochondrial function through suppression of α-Synuclein expression in cancer cells

**DOI:** 10.18632/oncotarget.16706

**Published:** 2017-03-30

**Authors:** Mai-Tram Vo, Seong Hee Choi, Ji-Heon Lee, Chung Hwan Hong, Jong Soo Kim, Unn Hwa Lee, Hyung-Min Chung, Byung Ju Lee, Jeong Woo Park, Wha Ja Cho

**Affiliations:** ^1^ Department of Biological Sciences, University of Ulsan, Ulsan, 680-749, Korea; ^2^ Department of Stem Cell Biology, School of Medicine, Konkuk University, Gwangjin-Gu, Seoul, 143-701, Korea

**Keywords:** tristetraprolin, mitochondrial dynamics, α-Synuclein

## Abstract

Mitochondrial dynamics play critical roles in maintaining mitochondrial functions. Here, we report a novel mechanism for regulation of mitochondrial dynamics mediated by tristetraprolin (TTP), an AU-rich element (ARE)-binding protein. Overexpression of TTP resulted in elongated mitochondria, down-regulation of mitochondrial oxidative phosphorylation, reduced membrane potential, cytochrome c release, and increased apoptotic cell death in cancer cells. TTP overexpression inhibited the expression of α-Synuclein (α-Syn). TTP bound to the ARE within the mRNA 3′-untranslated regions (3′-UTRs) of *α-Syn* and enhanced the decay of *α-Syn* mRNA. Overexpression of *α-Syn* without the 3′-UTR restored TTP-induced defects in mitochondrial morphology, mitochondrial oxidative phosphorylation, membrane potential, and apoptotic cell death. Taken together, our data demonstrate that TTP acts as a regulator of mitochondrial dynamics through enhancing degradation of *α-Syn* mRNA in cancer cells. This finding will increase understanding of the molecular basis of mitochondrial dynamics.

## INTRODUCTION

Mitochondria are involved in a variety of cellular functions [1]. Alterations in mitochondrial function are often associated with neurodegenerative disorders and cancer [2]. Depending on the cell type and physiological conditions, mitochondria can be present either as numerous morphologically small organelles, or they can form large interconnected networks [3–5]. Mitochondrial dynamics are determined by mitochondrial fusion and fission. Mitochondrial fusion and fission are mediated by three large GTPases and their interacting factors [6]. Fusion between mitochondrial outer membranes is mediated by the membrane-anchored dynamin family members mitofusin (Mfn)1 and Mfn2, whereas fusion between mitochondrial inner membranes is mediated by a single dynamin family member called optic atrophy 1 (OPA1) [6]. Fission is mediated by a cytosolic dynamin family member dynamin related protein 1 (Drp1). Mitochondrial fission protein 1 (Fis1) acts as receptors that recruit Drp1 to mitochondrial surface [7], often at sites where mitochondria make contact with the endoplasmic reticulum [8]. Drp1 induces mitochondrial fission by forming helical structures that wrap around mitochondria [9–11]. Impairments in mitochondrial fission and fusion cause breakdown of the mitochondrial network, loss of mitochondrial DNA (mtDNA), respiratory defects, and increases in reactive oxygen species (ROS), mitophagy, and apoptosis [12–16] and play a role in the pathogenesis of mitochondrial disease [17–20].

Alpha-synuclein (α-Syn) is abundantly expressed in the brain [21, 22] and is associated with the SNARE protein complex to facilitate vesicular trafficking and neurotransmitter release in the presynaptic terminal [23, 24]. α-Syn exists as both monomers and oligomers and forms β-sheet-rich α-Syn amyloid fibrils [25], which leads to neurodegenerative diseases such as Parkinson's (PD) and Alzheimer's disease [26–29]. α-Syn is also detected in peripheral cancers, including ovarian and breast [30], colorectal tumors [31], and melanoma [32]. In addition to its predominantly cytosolic and vesicular localization, α-Syn binds to the mitochondria [33–35] and leads to mitochondrial fragmentation when overexpressed by inhibiting membrane fusion [36]. Consistently, mitochondrial pathology has been reported in transgenic mice overexpressing wild-type or mutant α-Syn [37–39].

Post-transcriptional regulation of gene expression is mediated by AU-rich elements (AREs) located in the 3′-UTR of a variety of short-lived mRNAs such as cytokines and proto-oncogenes [40]. The destabilizing function of AREs is regulated by ARE-binding proteins [41]. One of the best-characterized ARE-binding proteins is tristetraprolin (TTP), which promotes degradation of ARE-containing transcripts [42–44]. TTP expression is significantly decreased in various cancers [45], which correlates with increased expression of proto-oncogenes and, as a result, may lead to abnormalities that contribute to cancer processes. Re-expression of TTP induces growth inhibitory effects [46–49].

In this study, we demonstrate for the first time that TTP expression alters mitochondrial morphology. Overexpression of TTP enhanced mitochondrial fusion in cancer cell lines. TTP did not increase the degradation of mRNAs from the three large GTPases involved in mitochondrial fission and fusion but did enhance mRNA degradation of *a-Syn*. Exogenous expression of *a-Syn* without the 3′-UTR recovered the mitochondrial morphology, suggesting that downregulation of *a-Syn* induces mitochondrial fusion. Down-regulation of *a-Syn* by TTP impaired mitochondrial functions, which decreased mitochondrial membrane potential, increased ROS production, induced apoptosis, and inhibited growth of cancer cells. Taken together, these findings suggest that TTP plays an important role as a regulator of mitochondrial dynamics through down-regulating expression of *a-Syn* in cancer cells.

## RESULTS

### TTP overexpression promotes an elongation of mitochondria

**Figure 1:**
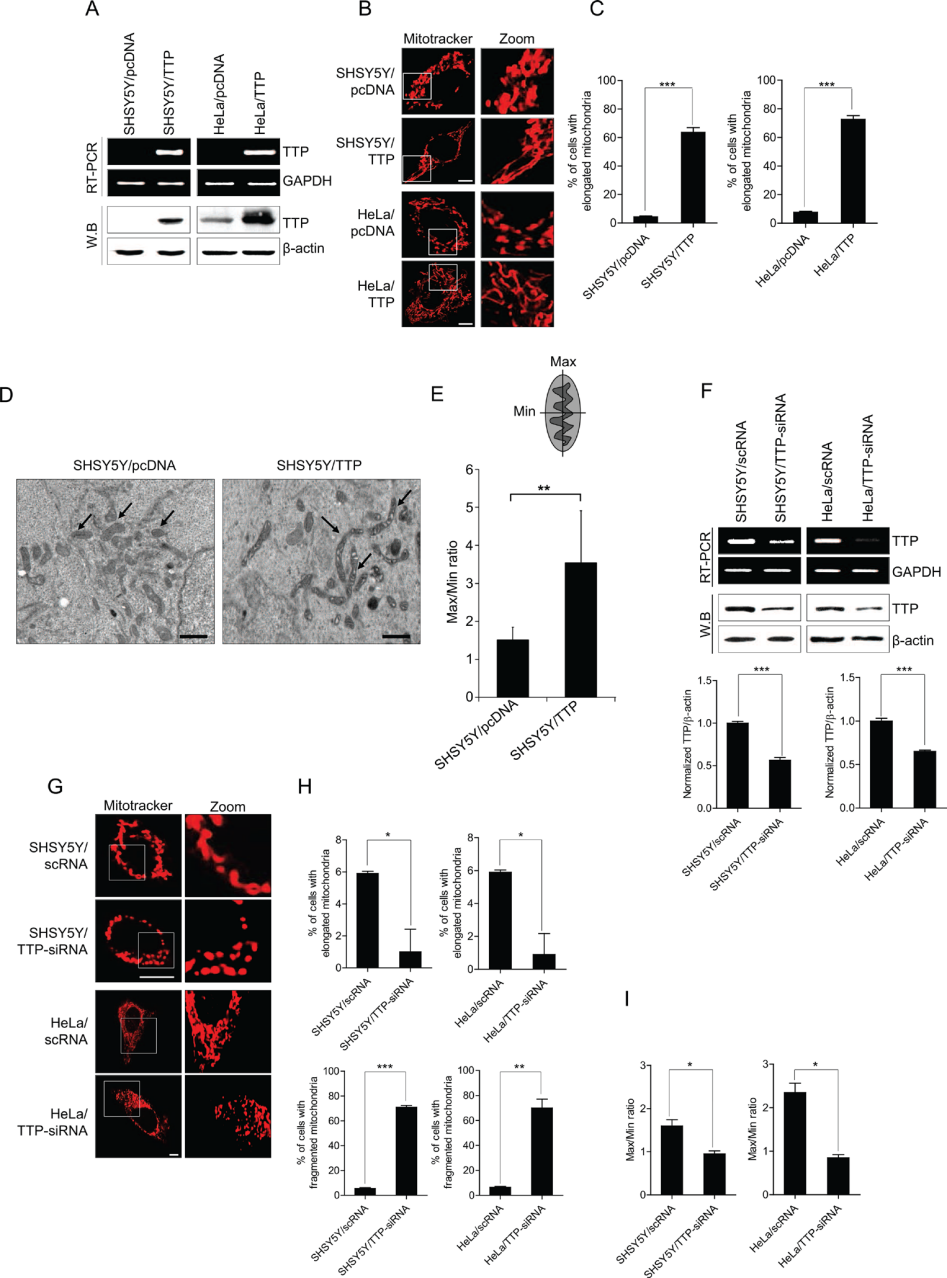
TTP overexpression induces mitochondrial elongation (**A**–**E**) SHSY5Y and/or HeLa cells were transiently transfected with pcDNA6/V5-TTP (SHSY5Y/TTP and HeLa/TTP) or with empty vector pcDNA6/V5 (SHSY5Y/pcDNA and HeLa/pcDNA) for 48 h. (A) TTP levels were determined by RT-PCR (top) and western blot (bottom). (B and C) Cells were stained with Mitotracker CMXRos for 30 min, and then images were obtained by confocal microscopy. (B) Representative confocal images with magnified insets of boxed areas. Scale bar, 10 μm. (C) Graphs represent percentage of cells with elongated mitochondria. Values are mean ± s.e.m. from three separate experiments with 100 cells per group per experiment (***p < 0.001). (D) Representative electron microscopic images of mitochondria. Scale bar, 1 μm. (E) Graphs represent ratio of maximum axis to minimum axis of mitochondria. Values are mean ± s.e.m. from three separate experiments (**p < 0.01). (**F**–**I**) TTP inhibition induced mitochondrial fragmentation. SHSY5Y and HeLa cells were transiently transfected with scRNA (SHSY5Y/scRNA and HeLa/scRNA) or TTP-siRNA (SHSY5Y/TTP-siRNA and HeLa/TTP-siRNA) for 48 h. (F) TTP levels were determined by RT-PCR (top) and western blot (bottom). The band densities in the western blot were quantified by Image J, normalized to the internal control β-actin and expressed as ratio of the value of control cells. Data shown are mean ± s.e.m. (*n* = 3). (***p < 0.001). (G–I) Cells were stained with Mitotracker CMXRos for 30 min, and images were obtained by confocal microscopy. (G) Representative confocal images with magnified insets of boxed areas. Scale bar, 10 μm. (H) Graphs represent percentage of cells with (top) elongated and (bottom) fragmented mitochondria. Values are mean ± s.e.m. from three separate experiments with 100 cells per group per experiment (*p < 0.05; **p < 0.01; (***p < 0.001). (I) Graphs represent ratio of maximum axis to minimum axis of mitochondria. Values are mean ± s.e.m. from three separate experiments (*p < 0.05).

Previously, we reported that overexpression of TTP suppresses cellular proliferation [46, 47, 50] and induces a change in cell morphology from a mesenchymal shape to an epithelial shape [51]. Here we assessed whether TTP overexpression modifies mitochondrial morphology. To test this, SHSY5Y neuroblastoma cells and HeLa cervical carcinoma cells were transiently transfected with pcDNA6/V5-TTP (SHSY5Y/TTP and HeLa/TTP) or a control pcDNA6/V5 (SHSY5Y/pcDNA and HeLa/pcDNA) vector. After confirming the overexpression of TTP by RT-PCR and western blot analysis (Figure 1A), mitochondria in the cells were stained with Mitotracker. Confocal microscopic imaging of mitochondria showed that TTP overexpression promoted the elongation of the mitochondrial compared with control cells in both SHSY5Y and HeLa cells (Figure 1B, 1C). To confirm this, mitochondrial morphology was observed using electron microscopy. Mitochondria of SHSY5Y cells overexpressing TTP demonstrated an elongated ultrastructure compared with control cells, and the average length of mitochondria in TTP-overexpressing cells was significantly increased over that of control cells (Figure 1D, 1E). We next tested whether down-regulation of TTP increased fragmentation of mitochondria. We used siRNA against *TTP* to reduce the expression level of TTP in SHSY5Y and HeLa cells. Down-regulation of *TTP* (Figure 1F) significantly increased fragmentation of mitochondria in both SHSY5Y and HeLa cells (Figure 1G–1I). Our results suggest that TTP plays an important role in the regulation of mitochondrial morphology.

### TTP does not decrease the expression of large GTPases involved in mitochondrial fusion and fission but does inhibit the expression of α-Syn

**Figure 2:**
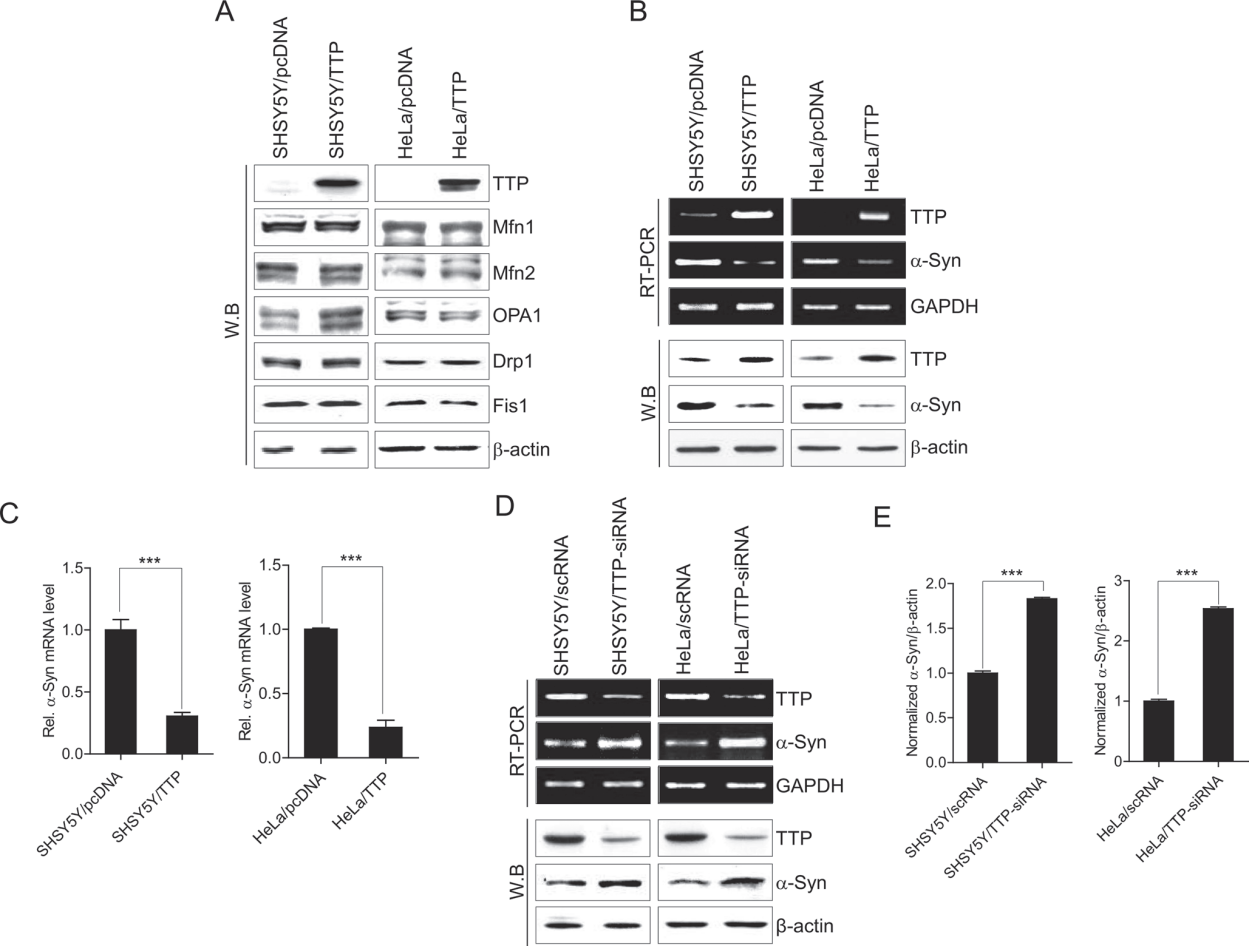
TTP overexpression does not inhibit Mfn1, Mfn2, OPA1, Drp1, and Fis1 expression levels but decreased α-Syn levels (**A**) TTP overexpression did not decrease the expression levels of Mfn1, Mfn2, OPA1, Drp1, and Fis1. SHSY5Y and HeLa cells were transiently transfected with pcDNA6/V5-TTP (SHSY5Y/TTP and HeLa/TTP) or empty vector pcDNA6/V5 (SHSY5Y/pcDNA and HeLa/pcDNA) for 48 h. Cells were analyzed for protein levels by western blot. (**B** and **C**) TTP overexpression decreased α-Syn levels. SHSY5Y and HeLa cells were transiently transfected with pcDNA6/V5-TTP (SHSY5Y/TTP and HeLa/TTP) or empty vector pcDNA6/V5 (SHSY5Y/pcDNA and HeLa/pcDNA) for 48 h. The levels of TTP and *α-Syn* were determined by RT-PCR (B, top), western blotting (B, bottom), and qRT-PCR (C). Values are mean ± s.e.m. from three separate experiments (***p < 0.001). (**D** and **E**) Inhibition of TTP increased α-Syn levels. SHSY5Y and HeLa cells were transiently transfected with scRNA (SHSY5Y/scRNA and HeLa/scRNA) or TTP-siRNA (SHSY5Y/TTP-siRNA and HeLa/TTP-siRNA) for 48 h. The levels of TTP and α-Syn were determined by RT-PCR (D, top) and western blot (D, bottom). (E) The band densities in the western blot were quantified by Image J, normalized to the internal control β-actin and expressed as ratio of the value of control cells. Values are mean ± s.e.m. from three separate experiments (***p < 0.001).

Mitochondrial morphology is regulated by mitochondrial dynamics, fusion, and fission [52]. Mfn1, Mfn2, and OPA1 have been identified for the mitochondrial fusion process, while Drp1, and Fis1 are thought to play critical roles in the fission process [53]. This prompted us to investigate whether TTP overexpression inhibits the expression of these mitochondrial fusion and fission proteins in SHSY5Y and HeLa cells. When we analyzed the expression levels of Mfn1, Mfn2, OPA1, Drp1, and Fis1 in SHSY5Y and HeLa cells by western blot, RT-PCR, and qRT-PCR, we unexpectedly found that overexpression of TTP did not decrease expression levels of these genes in either SHSY5Y or HeLa cells (Figure 2A).

Previously, it was reported that *α*-Syn decreased mitochondrial fusion [36] and enhanced mitochondrial fragmentation [54]. Analysis of *a-Syn* 3′-UTR revealed the presence of several AREs within its 3′-UTR (see below). Thus, we tested whether TTP overexpression inhibited *a-Syn* expression. Interestingly, we found that the expression levels of *a-Syn* were decreased in TTP-overexpressing SHSY5Y/TTP and HeLa/TTP cells (Figure 2B, 2C). To test whether down-regulation of TTP increased *a-Syn* expression, we used siRNA against *TTP* to reduce the expression level of TTP in SHSY5Y and HeLa cells. Down-regulation of *TTP* significantly increased the expression level of *a-Syn* (Figure 2D, 2E). These results suggest TTP is functionally involved in regulation of *a-Syn* expression.

### Overexpression of *a-Syn* attenuates TTP-induced changes in mitochondrial morphology

**Figure 3:**
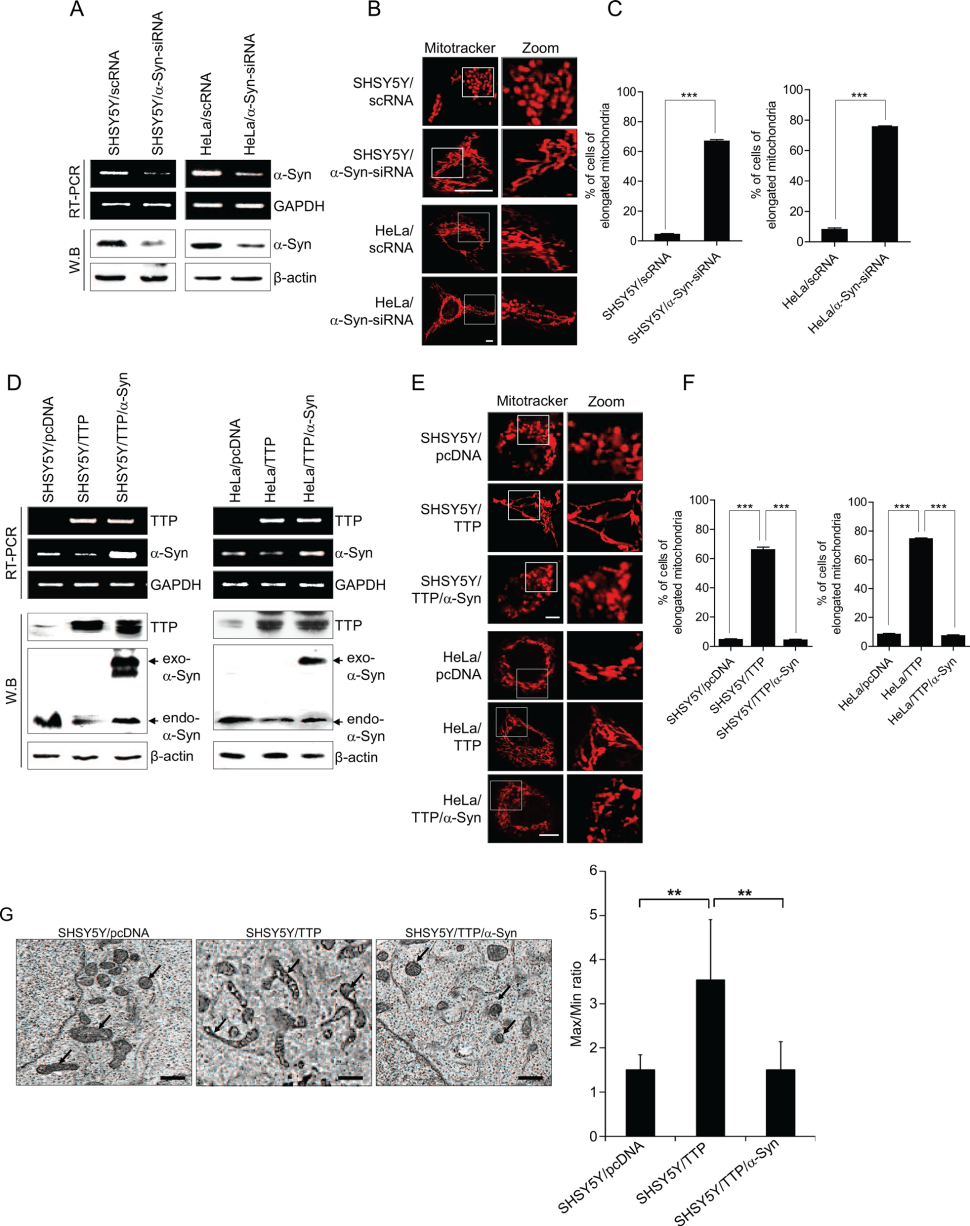
Overexpression of *α-Syn* inhibits mitochondrial elongation induced by TTP overexpression (**A**–**C**) Inhibition of α-Syn induced elongation of mitochondria. SHSY5Y and HeLa cells were transiently transfected with scRNA (SHSY5Y/scRNA and HeLa/scRNA) or α-Syn-siRNA (SHSY5Y/α-Syn-siRNA and HeLa/α-Syn-siRNA) for 48 h. (A) The levels of α-Syn determined by RT-PCR (top) and western blot (bottom). (B and C) Cells were stained with Mitotracker CMXRos for 30 min, and images were obtained by confocal microscopy. (B) Representative confocal images with magnified insets of boxed areas. Scale bar, 10 μm. (C) Graphs represent percentage of cells with elongated mitochondria. Values are mean ± s.e.m. from three separate experiments with 100 cells per group per experiment (***p < 0.001). (**D**–**G**) Transfection of *α-Syn* cDNA without the 3′-UTR abolished the effects of TTP on mitochondrial morphology. SHSY5Y and/or HeLa cells transfected with a combination of pcDNA6/V5-TTP and pcDNA/α-Syn for 48 h. Cells were stained with Mitotracker CMXRos for 30 min, and images were obtained by confocal microscopy. (D) The levels of TTP and α-Syn were measured by RT-PCR (top panel) and western blot assays (bottom panel). (E) Representative confocal images with magnified insets of boxed areas. Scale bar, 10 μm. (F) Graphs represent percentage of cells with elongated mitochondria. Values are mean ± s.e.m. from three separate experiments with 100 cells per group per experiment (***p < 0.001). (G) Representative electron microscopic images of mitochondria. Scale bar, 1 μm. (E) Graphs represent ratio of maximum axis to minimum axis of mitochondria. Values are mean ± s.e.m. from three separate experiments (*p < 0.05; **p < 0.01).

TTP overexpression decreased the expression level of *a-Syn* (Figure 2B, 2C) and induced the elongation of mitochondria (Figure 1A–1D) indicating the possibility that down-regulation of *a-Syn* by TTP contributed to the elongation of mitochondria in TTP-overexpressing cells. We first determined whether mitochondrial morphology could be modulated by the expression level of *a-Syn* in SHSY5Y and HeLa cells. Consistent with previous reports [36, 54], inhibition of *a-Syn* expression by siRNA (Figure 3A) increased mitochondrial length (Figure 3B, 3C and Supplementary Figure 1) and, on the contrary, overexpression of *a-Syn* decreased the length of mitochondria (Supplementary Figure 2). Next, we determined whether overexpression of *a-Syn* which did not contain a 3′-UTR restored the mitochondrial morphology in SHSY5Y/TTP and HeLa/TTP cells. To test this, SHSY5Y and HeLa cells were co-transfected with pcDNA6/V5-TTP and pcDNA/α-Syn. Overexpression of α*-Syn* (Figure 3D) significantly inhibited the elongation of mitochondria induced by TTP overexpression (Figure 3E–3G). These results indicate that TTP overexpression induced elongation of mitochondria through the down-regulation of *a-Syn* in SHSY5Y and HeLa cells.

### TTP destabilizes *a-Syn* mRNA

**Figure 4:**
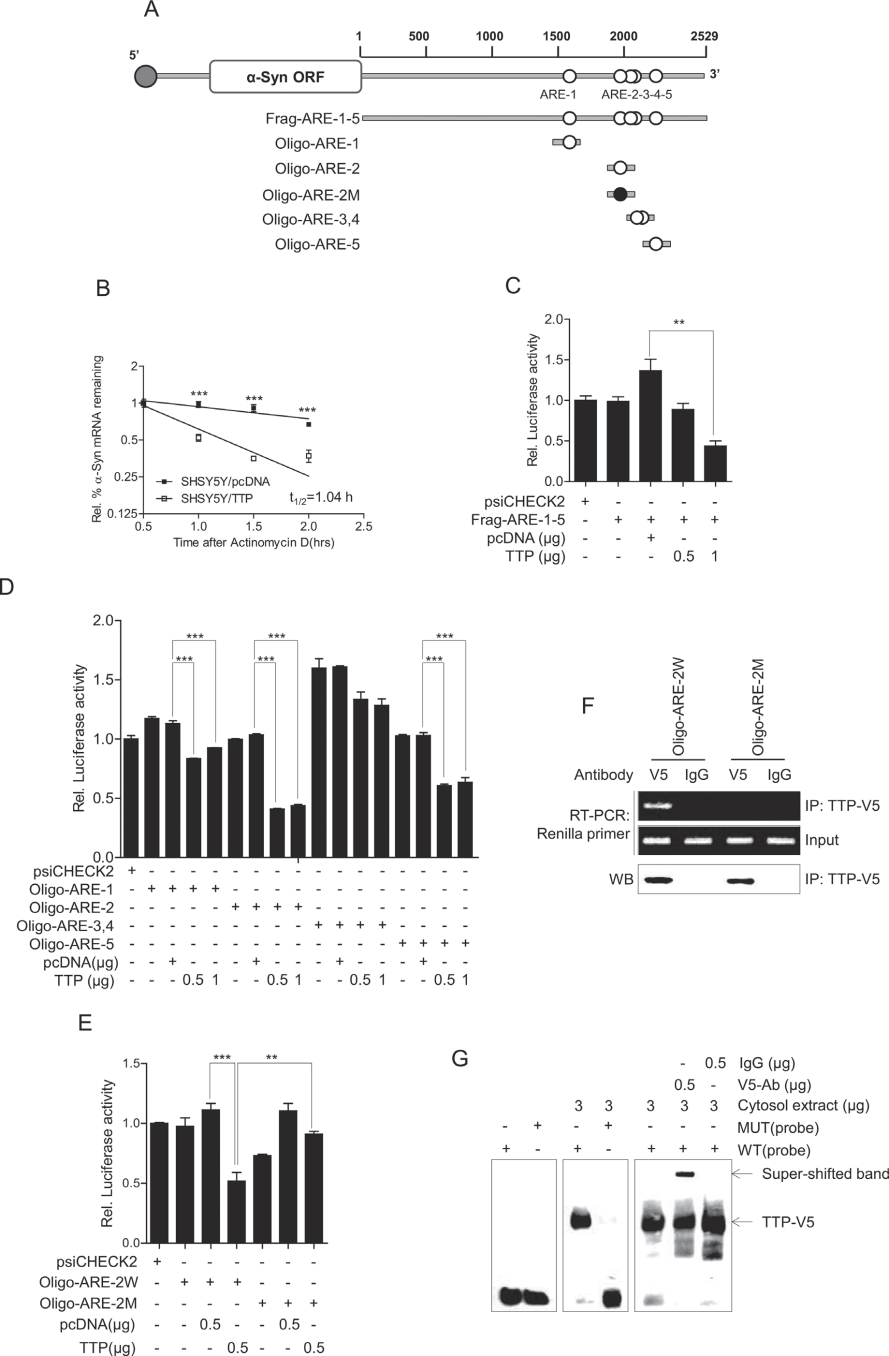
TTP enhances the decay of *α-Syn* mRNA by binding with AREs within the mRNA 3′-UTRs (**A**) Schematic representation of the luciferase reporter constructs used in this study. (**B**) TTP destabilized *α-Syn* mRNAs. 2 × 10^6^ SHSY5Y cells were transfected with 6 μg pcDNA6/V5-TTP or pcDNA6/V5 for 24 h. The expression of *α-Syn* mRNAs in SHSY5Y cells was determined by qRT-PCR at the indicated times after addition of 5 μg/ml actinomycin D. Data are presented as the mean *±* s.e.m. (*n* = 3) (***p < 0.001). (**C**–**E**) The second AUUUA pentamer (ARE-2) within the *α-Syn* 3′-UTR is necessary for the inhibitory effect of TTP. Fragments and oligonucleotides (Oligo) derived from the *α-Syn* mRNA 3′-UTR were cloned downstream of the luciferase reporter gene in the psiCHECK2 luciferase expression vector. White circles, wild-type (WT) pentameric motif AUUUA; black circles, mutated (MUT) motif AGCA. SHSY5Y cells were co-transfected with pcDNA6/V5-TTP and psiCHECK2 luciferase reporter constructs containing fragments or oligonucleotides derived from the *α-Syn* mRNA 3′-UTR as described in (A) for 24 h. After normalizing for luciferase activity, the luciferase activity obtained from the SHSY5Y cells transfected with the psiCHECK2 vector alone were set to 1.0. Data are presented as the mean *±* s.e.m. (*n* = 3) (**p < 0.01; ***p < 0.001). (**F**) Ribonucleoprotein immunoprecipitation assay. SHSY5Y cells were cotransfected with pcDNA6/V5-TTP and psiCHECK2 luciferase reporter constructs containing *α-Syn* Oligo-ARE-2W. The psiCHECK2 luciferase reporter construct containing mutant ARE-2, Oligo-ARE-2M was used as a negative control. At 48 h after transfection, the ribonucleoprotein complexes containing TTP were immunoprecipitated with protein G-agarose and anti-V5 or a control antibody. Luciferase mRNA in the immunoprecipitates was amplified by RT-PCR. The presence of TTP in the immunoprecipitates was detected by western blot with anti-V5 antibody. (**G**) RNA EMSA was performed by mixing cytoplasmic extracts containing 5 μg of total protein from pcDNA6/V5-TTP-transfected SHSY5Y cells with 20 fmol biotinylated wild-type Oligo-ARE-2W (WT) or mutant Oligo-ARE-2M (MUT) probe. Anti-TTP or control antibody was added to the reaction mixtures. The positions of the TTP-containing bands (TTP) are indicated.

The TTP protein decreases mRNA stability through binding to the AREs within the mRNA 3′-UTR [42, 43, 46, 47, 55]. Analysis of the 2529-bp-long human *a-Syn* 3′-UTR revealed the presence of five pentameric AUUUA (ARE) motifs (Figure 4A). To determine whether TTP-induced inhibition of α-Syn expression resulted from decrease in the stability of *a-Syn* mRNA, the half-life of *a-Syn* mRNA was measured by qRT-PCR in SHSY5Y cells transfected with pcDNA6/V5-TTP or with the pcDNA6/V5 control vector. In the control SHSY5Y/pcDNA cells, the half-life of *a-Syn* mRNA was >2 h after actinomycin D treatment. However, in TTP-overexpressing cells, the half-life was reduced to 1 h in SHSY5Y/TTP cells (Figure 4B). These results indicated that overexpression of TTP contributed to a decrease in α-Syn levels through the destabilization of *a-Syn* mRNA.

To determine whether down-regulation of *a-Syn* expression by TTP was mediated through the *a-Syn* mRNA 3′-UTR, we used a luciferase reporter gene linked to the *a-Syn* 3′-UTR fragment containing all five AREs, Frag-ARE-1-5, in the plasmid psiCHECK2. When SHSY5Y cells were transfected to overexpress TTP, luciferase activity was inhibited (Figure 4C). We next determined which AREs within the *a-Syn* 3′-UTR were important for TTP activity. We prepared a luciferase reporter gene linked to oligonucleotides containing each ARE within the *a-Syn* 3′-UTR (Oligo-ARE-1, Oligo-ARE-2, Oligo-ARE-3,4, and Oligo-ARE-5) in the plasmid psiCHECK2. In SHSY5Y cells overexpressing TTP, the luciferase activity of Oligo-ARE-2 was inhibited by 60% (Figure 4D), while the other Oligo-AREs did not respond or only slightly responded. To determine the importance of the second ARE (ARE-2), we prepared luciferase reporter genes containing wild-type (Oligo-ARE-2W, containing wild-type ARE-2) and mutant (Oligo-ARE-2M, containing AUUUA sequences substituted with AGCA) oligonucleotides. While Oligo-ARE-2W responded to TTP (60% inhibition), Oligo-ARE-2M did not respond to TTP (Figure 4E). These results suggested that the second ARE within the *a-Syn* mRNA 3′-UTR was involved in TTP inhibitory activity.

### TTP binds to the second ARE within the *a-Syn* mRNA 3′-UTR

To determine whether TTP interacted with ARE-2 of the *a-Syn* 3′-UTR, SHSY5Y cells were co-transfected with pcDNA6/V5-TTP and psiCHECK2-Oligo-ARE-2W (Oligo-ARE-2W) or psiCHECK2-Oligo-ARE-2M (Oligo-ARE-2M). After immunoprecipitation with anti-V5 or control antibody (IgG), the presence of TTP was determined by western blot analysis using an anti-V5 antibody (Figure 4F). Total RNA was extracted from the immunoprecipitates, and the presence of luciferase mRNA was analyzed by RT-PCR using PCR primers specific to the luciferase gene. The amplified PCR product was observed in immunoprecipitates from cells transfected with Oligo-ARE-2W and pcDNA6/V5-TTP (Figure 4F). However, no PCR products were detected in samples from cells transfected with Oligo-ARE-2M or pcDNA6/V5-TTP (Figure 4F). PCR product was not also detected in immunoprecipitates obtained using control antibody. These results demonstrated that TTP interacted specifically with the *a-Syn* ARE-2.

To confirm the interaction of TTP with ARE-2 of the *a-Syn* 3′-UTR, RNA EMSA was conducted using a biotinylated RNA probe containing wild-type or mutant ARE-2 of *a-Syn*. The RNA probes used for RNA EMSA were the same as those for the luciferase assay. Cytoplasmic extracts were prepared from SHSY5Y cells transfected with pcDNA6/V5-TTP to overexpress TTP and were incubated with a biotinylated RNA probe containing wild-type or mutant ARE-2 of *a-Syn*. When RNA EMSA was conducted using the wild-type ARE-2 probe of *a-Syn*, a dominant probe-protein complex was observed. However, the mutant ARE-2 of *a-Syn* prevented the formation of this complex. The complex was supershifted with the anti-V5 antibody (Figure 4G). These results confirmed that *a-Syn* ARE-2 was essential for TTP binding. Taken together, these data strongly suggest that repression of *a-Syn* occurs through binding of TTP to ARE-2 of *a-Syn* 3′-UTR.

### TTP overexpression decreases mitochondrial membrane potential and ATP production

**Figure 5:**
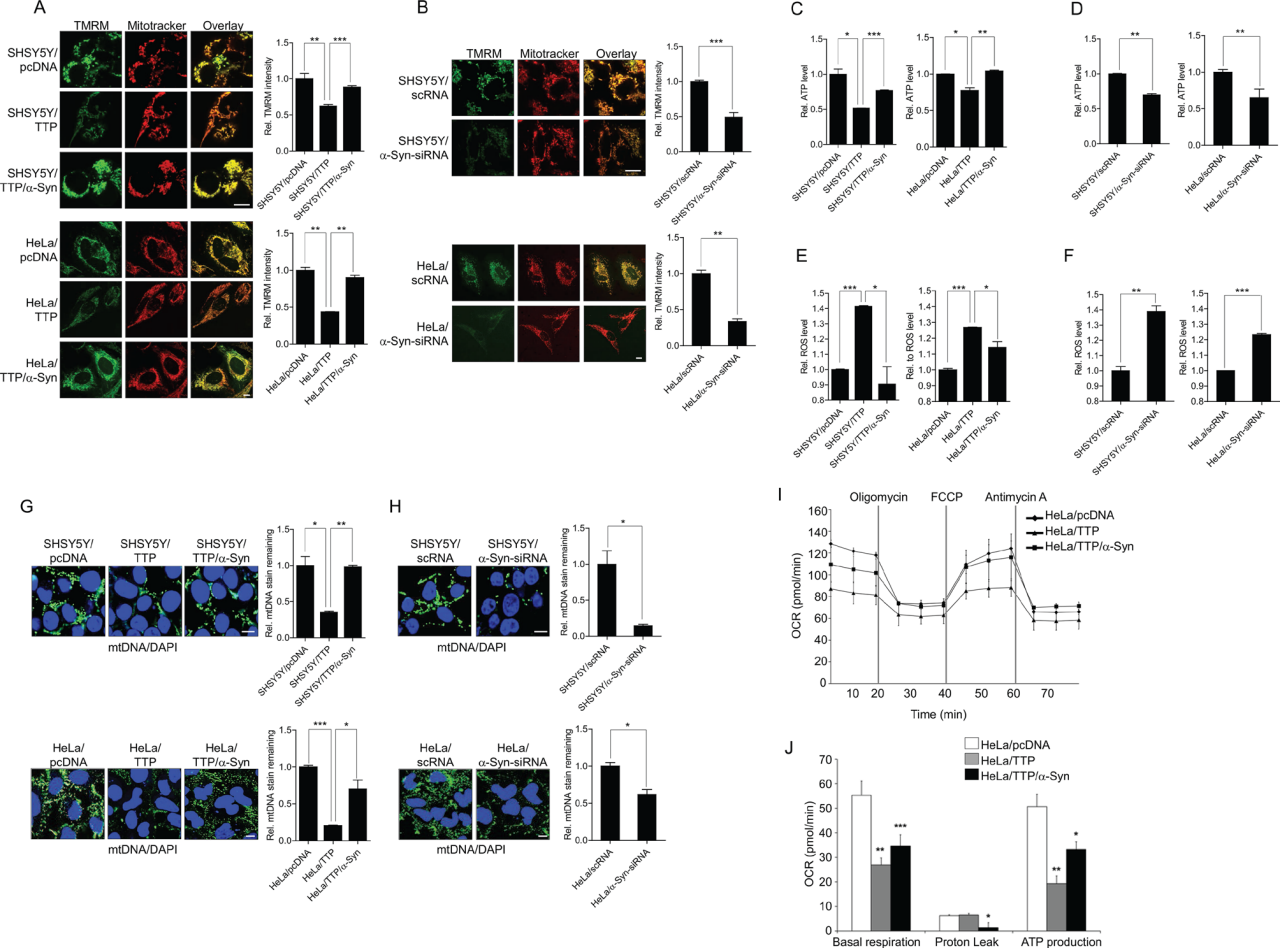
TTP overexpression induces mitochondrial dysfunction (**A** and **B**) Effects of TTP overexpression on mitochondrial membrane potential. SHSY5Y and HeLa cells were transfected with (A) a combination of pcDNA6/V5-TTP and pcDNA/α-Syn or (B) scRNA or α-Syn-siRNA for 48 h. Cells were stained with TMRM (pseudo-green) and Mitotracker CMXRos (red) for 30 min, and images were obtained by confocal microscopy. TMRM intensity reflects mitochondrial membrane potential level. The panels display representative confocal images with magnified insets of boxed areas. Scale bar, 10 μm. Graphs represent relative TMRM intensity. Values are mean ± s.e.m. from three separate experiments with 15 cells per group per experiment (**p < 0.01; ***p < 0.001). (**C** and **D**) Effects of TTP overexpression on ATP production. Cellular ATP levels were measured in SHSY5Y and HeLa cells transfected with (C) a combination of pcDNA6/V5-TTP and pcDNA/α-Syn or (D) scRNA or *α-Syn*-siRNA using the CellTiter-Glo luminescent cell viability assay. Values are means ± s.e.m. from three independent experiments (*p < 0.05; **p < 0.01; ***p < 0.001). (**E** and **F**) Effects of TTP overexpression on ROS production. ROS production was measured in SHSY5Y and HeLa cells transfected with (E) a combination of pcDNA6/V5-TTP and pcDNA/α-Syn or (F) scRNA or *α-Syn*-siRNA using the ROS-Glo^™^ H_2_O_2_ Assay Kit. Values are means ± s.e.m. of three independent experiments (*p < 0.05; **p < 0.01; ***p < 0.001). (**G** and **H**) Effects of TTP overexpression on mtDNA. SHSY5Y and HeLa cells were transfected with (G) a combination of pcDNA6/V5-TTP and pcDNA/α-Syn or (H) scRNA or *α-SYN*-siRNA for 48 h. Cells were stained with anti-DNA antibody for mtDNA (green) and DAPI for nuclear DNA (blue) as described in Materials and Methods. The panels display representative confocal images. Graphs represent relative percentage of mtDNA stain remaining. Values are mean ± s.e.m. from three separate experiments with 100 cells per group per experiment (*p < 0.05; **p < 0.01; ***p < 0.001). Scale bar, 10 μm. (**I** and **J**) TTP overexpression inhibits oxygen consumption rate (OCR). HeLa cells were transfected with a combination of pcDNA6/V5-TTP and pcDNA/α-Syn for 48 h. OCR of cells were measured by the XF-96 Extracellular Flux Analyzer. OCR was measured under basal conditions followed by the sequential addition of oligomycin (1 μM), FCCP (1 μM) or antimycin A (1 μM). Each data point represents an OCR measurement. (I) Representative traces of OCR measurements. (J) Graphs represent basal respiration, proton leak, and ATP production. Values are mean ± s.e.m. from three separate experiments (*p < 0.05; **p < 0.01; ***p < 0.001).

Imbalances in mitochondrial fission/fusion have been linked to mitochondrial dysfunction such as a decrease in mitochondrial membrane potential and OXPHOS, loss of mitochondrial DNA (mtDNA), increase in ROS production, and release of cytochrome c [56–63], leading to defects in energy production and induction of apoptosis. To investigate whether elongation of mitochondria in TTP-overexpressing cells was associated with mitochondrial dysfunction, we first examined mitochondrial membrane potential using the dye tetramethylrhodamine methyl ester (TMRM). TTP overexpression caused an approximately 38% and 57% decrease in green fluorescence in SHSY5Y and HeLa cells, respectively, indicating a marked loss of mitochondrial membrane potential (Figure 5A). Inhibition of *a-Syn* by using *a-Syn*-siRNA decreased mitochondrial membrane potential in control SHSY5Y and HeLa cells (Figure 5B), and overexpression of *α-Syn* by transfection with *α-Syn* without mRNA 3′-UTR reversed the decrease in mitochondrial membrane potential in TTP-overexpressing SHSY5Y and HeLa cells (Figure 5A). Mitochondrial membrane potential (Δψm) is critical for maintaining respiratory chain physiological function to generate ATP. Consistent with the decrease in mitochondrial membrane potential, total ATP production was decreased by TTP overexpression or inhibition of *α-Syn* in both SHSY5Y and HeLa cells (Figure 5C, 5D). This phenomenon was reversed by co-transfection with *α-Syn* without mRNA 3′-UTR (Figure 5C). Thus, these results suggest the elongation of mitochondria induced by TTP overexpression was accompanied by mitochondrial depolarization and loss of ATP production, which were reversed by overexpression of *α-Syn*.

### TTP overexpression increases ROS generation and decreases mtDNA contents

Mitochondrial dysfunction is often associated with increased ROS production by mitochondria [64] and ER [65]. ROS levels were significantly increased in SHSY5Y and HeLa cells in which *α-Syn* level was down-regulated by transient transfection with pcDNA6/V5-TTP (Figure 5E). Similarly, inhibition of *α-Syn* by using siRNA increased ROS levels in both SHSY5Y and HeLa cells (Figure 5F). Overexpression of *α-Syn* by transfection with *α-Syn* without the mRNA 3′-UTR significantly attenuated increased ROS levels induced by TTP overexpression in SHSY5Y and HeLa cells (Figure 5E).

mtDNA is located in close proximity to the respiratory chain, the main cellular source of ROS. Generation of ROS induces degradation of mtDNA [66]. Thus, we investigated whether ROS induced by TTP overexpression decreased mtDNA content. Overexpression of TTP or inhibition of *a-Syn* significantly decreased mtDNA content as quantified by immunofluorescence in both SHSY5Y and HeLa cells (Figure 5G, 5H). Transfection of *α-Syn* without mRNA 3′-UTR reversed the decrease in mtDNA content in TTP-overexpressing SHSY5Y and HeLa cells (Figure 5G).

### TTP overexpression decreases oxygen consumption

We further characterized mitochondrial dysfunction induced by TTP overexpression by analyzing oxygen consumption rate (OCR). SHSY5Y and HeLa cells overexpressing TTP showed decreases in basal respiration rates compared to control cells (Figure 5I, 5J). We also measured OCR after sequential addition of the ATP synthase inhibitor oligomycin, the uncoupler FCCP, the RC complex I inhibitor rotenone, and the RC complex III inhibitor antimycin A. TTP overexpression decreased ATP production (coupled respiration, the drop in the OCR after addition of oligomycin) in HeLa cells (Figure 5I, 5J). However, TTP overexpression did not affect the proton leak (uncoupled respiration, the difference between oligomycin- and rotenone and antimycin A-responsive OCRs) in HeLa cells (Figure 5I, 5J). Transfection of *α-Syn* without mRNA 3′-UTR reversed the decrease in basal respiration and ATP production in TTP-overexpressing HeLa cells (Figure 5I, 5J). We concluded from these results that TTP overexpression decreased respiration rate.

### TTP overexpression promotes apoptosis in cancer cells

**Figure 6:**
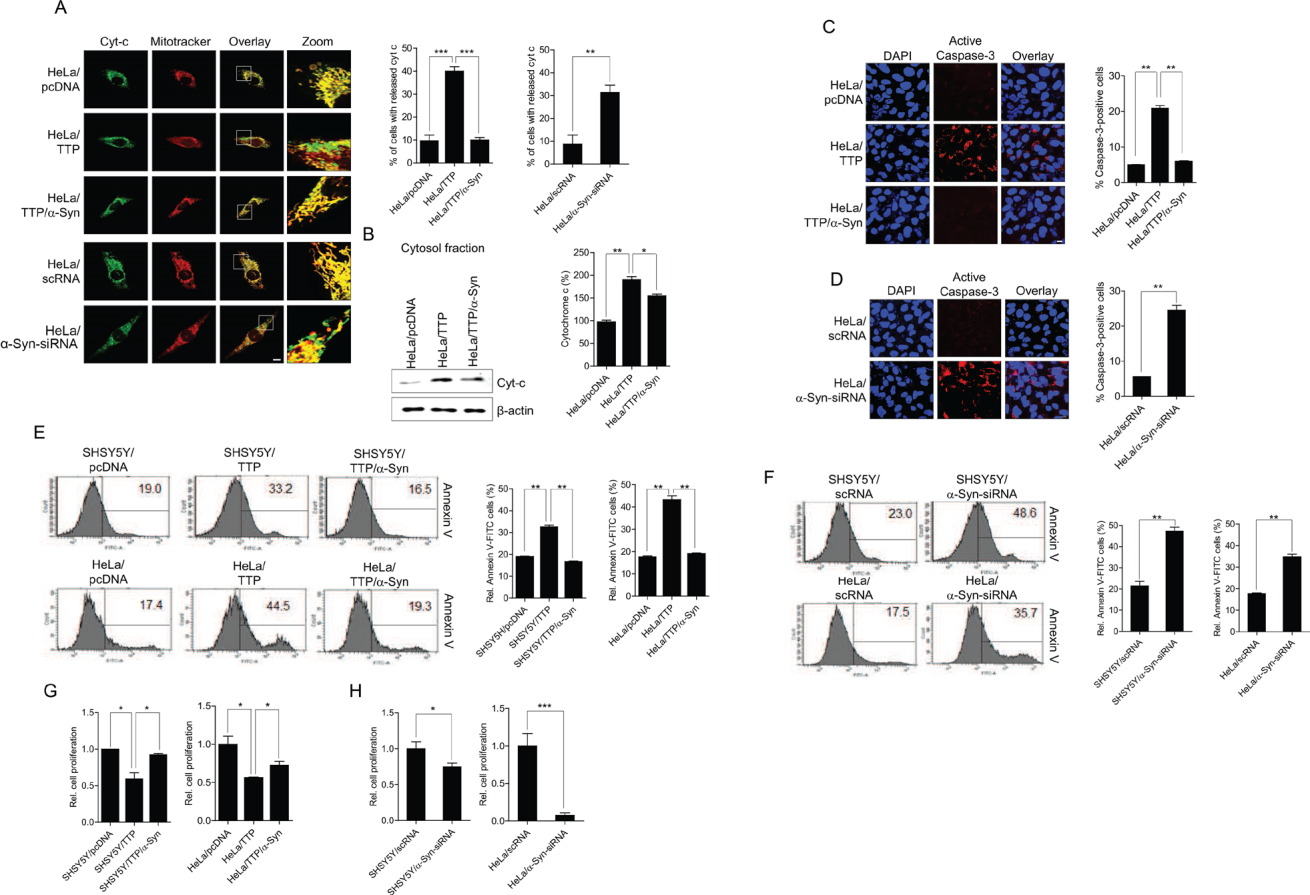
TTP overexpression increases cytochrome c release from mitochondria, caspase-3 activity and apoptosis in cancer cells (**A** and **B**) Effects of TTP overexpression on cytochrome release. HeLa cells were transfected with a combination of pcDNA6/V5-TTP and pcDNA/α-Syn for 48 h. Cells were stained with Mitotracker CMXRos (red) and anti-cytochrome c (green), then images were obtained by confocal microscopy. (A) Representative confocal images with magnified insets of boxed areas. Scale bar, 10 μm. (B) The cytosolic fraction of cells was analyzed for cytochrome c by western blot analysis. Graphs represent relative percentage of cytosolic cytochrome c. Values are mean ± s.e.m. from three separate experiments (*p < 0.05; **p < 0.01). (**C** and **D**) Effects of TTP overexpression on caspase-3 activity. HeLa cells were transfected with (C) a combination of pcDNA6/V5-TTP and pcDNA/α-Syn or (D) scRNA or *α-Syn*-siRNA for 48 h. Cells were analyzed for active caspase-3 using the Image-iT LIVE Red Caspase Detection Kit. Blue, DAPI. Scale bar, 10 μm. Graphs represent percentage of caspase-3-positive cells. Values are mean ± s.e.m. from three separate experiments with 100 cells per group per experiment (**p < 0.01). (**E** and **F**) Effects of TTP overexpression on apoptosis. HeLa cells were transfected with (E) a combination of pcDNA6/V5-TTP and pcDNA/α-Syn or (F) scRNA or *α-Syn*-siRNA for 48 h. Apoptotic cells were analyzed with Annexin V staining by flow cytometric analysis. The panels display representative histogram (**p < 0.01). (**G** and **H**) Effects of TTP overexpression on cell proliferation. SHSY5Y and HeLa cells were transfected with (G) a combination of pcDNA6/V5-TTP and pcDNA/α-Syn or (H) scRNA or *α-Syn*-siRNA for 48 h. Cells were seeded in triplicate in 24-well culture plates at 5 × 10^4^ cells/well and incubated for 24 h. Cells were then stained by Trypan-Blue Solution (0.4%) (T8154, Sigma) and counted using a hematocytometer. Graphs represent relative cell proliferation. Values are mean ± s.e.m. from three separate experiments (*p < 0.05; ***p < 0.001).

A reduction in mitochondrial membrane potential can induce caspase-dependent apoptosis by release of cytochrome c [67]. We thus determined whether a decrease in the mitochondrial membrane potential induced by TTP overexpression lead to cytochrome c release and apoptosis in HeLa cells. Release of cytochrome c was assessed by confocal microscopic observation and subcellular fractionation of control and TTP-overexpressing HeLa cells. TTP overexpression induced cytochrome c release into the cytosolic fraction (Figure 6A, 6B) and increased caspase-3 activity (Figure 6C). Consistently, inhibition of *α-Syn* using *a-Syn*-siRNA led to an increase in cytochrome c release (Figure 6A) and caspase-3 activity (Figure 6D). Overexpression of *α-Syn* without mRNA 3′-UTR inhibited cytochrome c release (Figure 6A, 6B) and caspase-3 activity (Figure 6C) in TTP-overexpressing cells. Next, apoptotic cells were detected by Annexin V staining. TTP overexpression or inhibition of *α-Syn* using *a-Syn*-siRNA increased apoptosis in both SHSY5Y and HeLa cells (Figure 6E, 6F) and expression of *α-Syn* without mRNA 3′-UTR reversed apoptosis in TTP-overexpressing cells (Figure 6E). Consistently, overexpression of TTP or inhibition of *α-Syn* using *a-Syn*-siRNA suppressed cell proliferation (Figure 6G, 6H), and expression of *α-Syn* without mRNA 3′-UTR rescued cell proliferation in TTP-overexpressing SHSY5Y and HeLa cells (Figure 6G). These results suggested that TTP overexpression induced cytochrome c release from mitochondria, activation of caspase-3, and apoptosis in cancer cells.

## DISCUSSION

The many functions of mitochondria are intimately linked to their morphology, which is maintained by mitochondrial dynamics [68, 69]. Here, we found that TTP regulated mitochondrial morphology and function: overexpression of TTP induced elongation of mitochondria and led to mitochondrial dysfunction and apoptosis. Mitochondrial dynamics and morphology are finely tuned by mitochondrial fusion and fission proteins Mfn1, Mfn2, OPA1, Drp1, and Fis1 [6, 7]. However, we found that TTP did not decrease the expression levels of the well-known mitochondrial fusion and fission proteins Mfn1, Mfn2, Drp1, and OPA1. Instead, TTP inhibited the expression of α-Syn, which lead to mitochondrial elongation and mitochondrial dysfunction. We here provide supporting evidence that 1) TTP binds to the 3′-UTR of *α-Syn* mRNA and enhances degradation of *α-Syn* mRNA, 2) transfection of *a-Syn* without mRNA 3′-UTR reverses mitochondrial morphology and 3) mitochondrial dysfunction is induced by TTP overexpression.

It is widely agreed that α-Syn expression levels are critical for the development of PD and that reducing α-Syn levels represents an attractive strategy to counteract the detrimental effects of the overexpression. Consistent with this hypothesis, overexpression of α-Syn results in severe neurodegeneration, suggesting a dose-dependent toxic effect [70, 71]. While accumulation of α-Syn by the inhibition of autophagy results in increased toxicity of α-Syn, reducing α-Syn levels by activation of autophagy has shown therapeutic effects in PD models [72–75]. Overexpression of α-Syn leads to mitochondrial fragmentation and neuronal cell death [36, 54], and reducing α-Syn levels by RNAi makes mitochondria more tubular in a neural cell line [36]. However, it is not likely that reduction of α-Syn levels always improves mitochondrial function and/or diseases. We here found that even though reducing α-Syn levels by TTP overexpression induces elongation of mitochondria, it also causes mitochondrial dysfunction including reduction in mitochondrial membrane potential and ATP generation, and cytochrome c release. Consistent with our results, it has been reported that robust reduction of α-Syn by siRNA increased neurotoxicity in rat models [76, 77] suggesting that either reducing or increasing the α-Syn level beyond the normal physiological level can cause defects in mitochondrial dynamics and mitochondrial dysfunction. Taken together, these results imply that regulation of α-Syn expression to maintain normal physiological levels is important for optimal mitochondrial function.

Besides in the central nervous system, α-Syn is expressed in a variety of tumors [30–32] and α-Syn expression has been proposed to affect tumorigenesis [78, 79]. These suggest the common pathogenic mechanisms between cancer and neurodegenerative diseases such as PD. Consistently, several studies have shown elevated risk for malignant melanoma in PD patients [80–82]. In this study, we found that TTP down-regulates α-Syn expression. Previously it has been reported that TTP exerts inhibitory effects on the growth of cancer cells [46–49]. Combining our results and previous reports, it is possible to suggest that the inhibitory effect of TTP on the growth of cancer cells may be partly mediated by down-regulation of α-Syn expression.

Expression of α-Syn is modulated by post-transcriptional control mechanisms targeting the 3′-UTR of mRNAs. Several reports have suggested that *α-Syn* mRNA levels are post-transcriptionally decreased by endogenous microRNAs (miRNAs) targeting the 3′-UTR of *α-Syn* mRNA. In rodent primary neurons, *α-Syn* levels are down-regulated by miR-7 and miR-153 [83]. In humans, miR-7, miR-34b and miR-34c target the 3′-UTR of *α-Syn* mRNA and reduce *α-Syn* mRNA levels, suggesting that down-regulation of miR-34b and miR-34c in the brain, as well as a single-nucleotide polymorphism (SNP) in the 3′-UTR of *α-Syn*, can increase α-Syn expression, possibly contributing to PD pathogenesis [84, 85]. Consistent with these studies, it has been reported that expression of these two miRNAs is down-regulated in human brains [86, 87]. In this study, we found TTP as a factor responsible for post-transcriptional regulation of *α-Syn* expression. TTP is an RNA-binding protein that binds to AU-rich elements within the mRNA 3′-UTR and inhibits gene expression through enhancing mRNA degradation [42–44]. TTP binds to a -AUUUA- motif within the 3′-UTR of *α-Syn* and inhibits expression of *α-Syn* through enhancing mRNA degradation. Mutation in this AUUUA motif blocks binding of TTP to the motif and, subsequently, the inhibitory effects of TTP. Based on our results, we predict that dysregulation of TTP or SNPs in the TTP target AUUUA motif within the 3′-UTR of *α-Syn* mRNA may increase α-Syn levels and cause mitochondrial dysfunction.

In conclusion, our data suggest that TTP could reduce the expression of α-Syn and cause defects in mitochondrial morphology and function. We demonstrated that TTP enhances *α-Syn* mRNA decay through binding to an AUUUA motif within the *α-Syn* mRNA 3′-UTR, and disruption of this AUUUA motif diminished the repressive activity of TTP. Considering the involvement of α-Syn in the pathological processes of neurodegenerative diseases such as PD and Alzheimer's disease [26–29] our present findings raise the possibility that TTP dysregulation and sequence variations in its target sequence within *α-Syn* mRNA 3′-UTR might trigger neurodegenerative diseases as well as mitochondrial dysfunction.

## MATERIALS AND METHODS

### Cell culture

Human cancer cell lines, SHSY5Y and HeLa were purchased from the Korean Cell Line Bank (KCLB-Seoul, Korea). SHSY5Y and HeLa cells were cultured in Dulbecco's Modified Eagle Medium (DMEM). All cell lines were supplemented with 10% FBS (heat-inactivated fetal bovine serum) (Welgene) and were maintained at 37°C in a humidified at atmosphere of 5% CO_2_.

### Plasmids, small interfering RNAs, transfections, and dual-luciferase assay

The pcDNA6/V5-TTP construct was described previously [46, 47]. The pcDNA/α-Syn construct was a gift from Wongi Seol (Wonkwang University Sanbon Hospital, Korea). SHSY5Y and HeLa cells were transfected with pcDNA6/V5-TTP or pcDNA/α-Syn using the TurboFect^™^ *in vitro* transfection reagent (R0531, Thermo Scientific).

Small interfering RNAs (siRNAs) against human α-Syn (*α-Syn*-siRNA, sc-29619), human TTP (*TTP*-siRNA, sc-36760) and control siRNA (scRNA, sc-37007) were purchased from Santa Cruz Biotechnology, Inc. (Santa Cruz, CA). SHSY5Y and HeLa cells were transfected 24 h after plating using Lipofectamine^™^ RNAiMAX (13778-150, Invitrogen) then were harvested at 48 h. The expression levels for *TTP* or *α-Syn* mRNA and protein were analyzed by RT-PCR or western blots.

A fragment of *α-Syn* 3′-UTR containing five pentameric AUUUA motifs was amplified from the cDNA of SHSY5Y cells using the following PCR primers: 5′- CCGCTCGAGCCTTAAAGGAGATCAATTCT-3′, 5′- AT AAGAATGCGGCCGCGCCACTTGGCAGGTGAAT GT-3′. The underlined sequences are restriction enzyme sites. PCR products were inserted into the XhoI/NotI sites of the psiCHECK2 Renilla/Firefly dual-luciferase expression vector (Promega) to generate psiCHECK2-*α-Syn* 3′-UTR. Four oligonucleotides containing the first AUUUA motif (Oligo-ARE-1), the second AUUUA motif (Oligo-ARE-2), the third and fourth AUUUA motifs (Oligo-ARE-3,4), and the fifth AUUUA motif (Oligo-ARE-5) within the *α-Syn* mRNA 3′-UTR were synthesized at ST Pharm. Co., Ltd. (Korea) (Table 1). These oligonucleotides were inserted into the XhoI/NotI sites of the psiCHECK2 expression vector (Promega). A mutant oligonucleotide in which the AUUUA pentamer in the second AUUUA motif was substituted with AGCA (Oligo-ARE-2M) was also synthesized. The oligonucleotide was ligated into the XhoI/NotI site of the psiCHECK2 vector.

**Table 1:** Oligonucleotides used to analyze α-Synuclein mRNA 3′-UTR

Oligonucleotides	Sequences
Oligo-ARE-1	F: 5′-TCGAGACTTGATGGTGAAAAACTCTGTATAAATTAATTTAAAAATTATTTGGTTTCTCTTTTTAATTATTGC-3′ R: 5′-GGCCGCAATAATTAAAAAGAGAAACCAAATAATTTTTAAATTAATTTATACA GAGTTTTTCACCATCAAGTC-3′
Oligo-ARE-2	F: 5′-TCGAGTAAATCTACCTAAAGCAGCATATTTTAAAAATTTAAAAGTATTGGTA TTAAATTAAGAAATAGAGGC-3′ R: 5′-GGCCGCCTCTATTTCTTAATTTAATACCAATACTTTTAAATTTTTAAAATATGC TGCTTTAGGTAGATTTAC-3′
Oligo-ARE-3,4	F: 5′-TCGAGAATTTGAGATTAGGAAAGTTGTGACCATGAATTTAAGGATTTATGT GGATACAAATTCTCCTTTAAAGTGTTTGC-3′ R: 5′-GGCCGCAAACACTTTAAAGGAGAATTTGTATCCACATAAATCCTTAAATTCA TGGTCACAACTTTCCTAATCTCAAATTC-3′
Oligo-ARE-5	F: 5′-TCGAGAATTCTCCTTTAAAGTGTTTCTTCCCTTAATATTTATCTGACGGTAATTTTTGAGCAGTGAATTACGC-3′ R: 5′-GGCCGCGTAATTCACTGCTCAAAAATTACCGTCAGATAAATATTAAGGGAA GAAACACTTTAAAGGAGAATTC-3′
Oligo-ARE-2M	F: 5′-TCGAGTAAATCTACCTAAAGCAGCATATTTTAAAAAGC AAAAGTATTGGTAT TAAATTAAGAAATAGAGGC-3′ R: 5′-GGCCGCCTCTATTTCTTAATTTAATACCAATACTTTTGCTTTTTAAAATAT GCTGCTTTAGGTAGATTTAC-3

Underlined sequences are restriction enzyme sites.

For the luciferase assays, cells were co-transfected with various psiCHECK2-*α-Syn* 3′-UTR constructs and pcDNA6/V5-TTP using the TurboFect^™^ *in vitro* transfection reagent (R0531, Thermo Scientific). Transfected cells were lysed with lysis buffer and mixed with luciferase assay reagent (017757, Promega), then the chemiluminescent signal was measured using a SpectraMax L Microplate reader (Molecular Devices). Firefly luciferase was normalized to Renilla luciferase in each sample. All luciferase assays reported here represent at least three independent experiments, each consisting of three wells per transfection.

### SDS-PAGE analysis and immunoblotting

Proteins were resolved by SDS-PAGE, transferred onto nitrocellulose membranes (10600001, GE Healthcare), and probed with appropriate dilutions of Anti-V5 Tag antibody (GWB-7DC53A, Genway Biotech), anti-TTP antibody (SAB4200505, Sigma), anti-α-Syn antibody (2642, Cell signaling), anti-Cytochrome-c antibody (4272, Cell signaling), anti-Caspase-3 antibody (9662, Cell signaling), anti-Drp1 antibody (8570, Cell signaling), anti-Mfn1 antibody (ab57602, Abcam), anti-Mfn2 antibody (ab56889, Abcam), anti-OPA1 antibody (ab42364, Abcam), anti-Fis1 antibody (S2229, Epitomics), and anti-actin (A5441, Sigma). Immunoreactivity was detected using the ECL detection system (GE Healthcare). Films were exposed at multiple time points to ensure that the images were not saturated. If required, the band densities were analyzed with NIH image software and normalized by comparison with the densities of internal control β-actin bands.

### RNA kinetics, quantitative real-time PCR, and RT-PCR

For RNA kinetic analysis, we used actinomycin D (A9415, Sigma) and assessed *α-Syn* mRNA expression using qRT-PCR. Briefly, 2 μg of total RNA was reverse transcribed using oligo-dT (79237, Qiagen) and MMLV reverse transcriptase (3201, Beamsbio) according to the manufacturer's instructions. qRT-PCR was performed by monitoring increase in fluorescence in real-time of the SYBR Green dye (MasterMix-R, Abm) using the StepOnePlus^™^ Real-time PCR system (Applied Biosystems). RT-PCR was performed using Taq polymerase 2X premix (Solgent) and appropriate primers. PCR primer pairs were as follows: TTP, 5′-CGCTACAAGACTGAGCTAT-3′ and 5′-GAGGTAGAACTTGTGACAGA-3′; α-Syn, 5′-TGTA GGCTCCAAAACCAAGG-3′ and 5′-TGTCAGGAT CCACAGGCATA-3′; Mfn1, 5′-TGTTTTGGTCGCA AACTCTG-3′ and 5′-CTGTCTGCGTACGTCTTCCA-3′; Mfn2, 5′-ATGCATCCCCACTTAAGCAC-3′ and 5′-CCA GAGGGCAGAACTTTGTC-3′; OPA1, 5′-TGTGAGG TCTGCCAGTCTTTA-3′ and 5′-TGTCCTTAATTGG GGTCGTTG-3′; Fis1, 5′-AGGCCGTGCTGAACGAG CTG-3′ and 5′-GGTAGTTCCCCACGGCCAGG-3′; Drp1, 5′-CACCCGGAGACCTCTCATTC-3′ and 5′-CCCCA TTCTTCTGCTTCCAC-3′; GAPDH, 5′-ACATCAAGA AGGTGGTGAAG-3′ and 5′-CTGTTGCTGTA GCCAAATTC-3′. mRNA half-life was calculated from non-linear regression of the mRNA level at 30-, 60-, 90-, and 120-min time points following addition of actinomycin D using GraphPad Prism 5.00 software based on a one-phase exponential decay model.

### Ribonucleoprotein immunoprecipitation (RNP) assay

RNP complexes were immunoprecipitated after reverse cross-linking between target RNA and proteins as described previously (47). Briefly, 1 × 10^7^ SHSY5Y cells were co-transfected with 10 μg of pcDNA6/V5-TTP and psiCHECK2-*α-Syn*-Oligo-ARE-2W or psiCHECK2-*α-Syn*-Oligo-ARE-2M. At 24 h after transfection, the cell suspension was incubated in 1% formaldehyde for 20 min at room temperature. The reaction was stopped with 0.25 M glycine (pH 7.0), and cells were sonicated in modified radioimmune precipitation assay buffer containing protease inhibitors (Roche Applied Science). RNP complexes were immunoprecipitated using protein G-agarose beads preincubated with 1 μg of anti-V5 Tag antibody (GWB-7DC53A, Genway Biotech) or 1 μg of isotype control (Sigma). After cross-linking reversion at 70°C for 45 min, RNA was isolated from the immunoprecipitates and treated with DNase I (Qiagen). cDNA was synthesized from the RNA, and the *Renilla* luciferase gene was amplified by PCR using *Taq* polymerase and *Renilla* luciferase-specific primers (Up, 5′-ACGTGCTGGACTCCTTCATC-3′; and Down, 5′-GACACTCTCAGCATGGACGA-3′). TTP proteins in the immunoprecipitated samples were detected by western blot analysis using anti-V5 Tag antibody.

### Electrophoretic mobility shift assay (EMSA)

Biotinylated RNA probes for the wild type (*α-Syn*-ARE-2W, 5′- UAAAUCUACCUAAAGC AGCAUA UUUUAAAAAUUUAAAAGUAUUGGUAUUAAAUU AAGAAAUAGAG-3′) and mutant (*α-Syn*-ARE-2M, 5′- UAAAUCUACCUAAAGCAGCAUAUUUUAAAAA GCAAAAGUAUUGGUAUUAAAUUAAGAAAUAGA G-3′) constructs were generated by ST Pharm. Co., Ltd. (Korea). A mutant RNA probe in which two AUUUA pentamers were each substituted with AGCA was used as a negative control. Cytoplasmic extracts were prepared from SHSY5Y cells and TTP-transfected SHSY5Y cells using NE-PER nuclear and cytoplasmic extraction reagent (78833, Thermo Scientific). An electrophoretic mobility shift assay (EMSAs) was performed using the LightShift chemiluminescent EMSA kit (20158, Thermo Scientific) according to the manufacturer's instructions. Briefly, 20 fmol of biotinylated RNA was combined with 4 μg of cytoplasmic protein from cell extract in binding buffer. For the supershift EMSA, rabbit anti-human TTP polyclonal antibody (ab36558, Abcam) or control antibody (I-5381, Sigma) was added to the reaction mixture. After the addition of antibodies, reaction mixtures were incubated on ice. The reaction mixtures were resolved on 5% non-denaturing polyacrylamide gels in 0.5× Tris borate/ EDTA buffer. Gels were transferred to nylon membrane (Hybond^™^-N) in 0.5× Tris borate/EDTA at 70 V for 40 min. Transferred RNAs were cross-linked to the membrane and detected using horseradish peroxidase-conjugated streptavidin (LightShift chemiluminescent EMSA kit, Thermo Scientific) according to the manufacturer's instructions.

### Annexin V staining

Annexin V staining was conducted using an Annexin-V-FLUOS staining kit, according to the protocol supplied by the manufacturer (11858777001, Roche). Briefly, cells were washed twice with PBS (137 mM NaCl, 2.7 mM KCl, 4.3 mM Na_2_HPO_4_. 7H_2_O, 1.4 mM KH_2_PO_4_, pH 7.2) and resuspended with binding buffer (0.01 M HEPES pH 7.4, 0.14 M NaCl, 2.5 mM CaCl_2_) containing Annexin V. Cells were analyzed for fluorescence intensity using a FACS flow cytometer (Becton Dickinson).

### Cell proliferation

Cells were transfected with a combination of pcDNA6/V5-TTP and pcDNA/α-Syn or with scramble or *α-Syn*-siRNA for 48 h. Cells were seeded in triplicate in 24-well culture plates at 5 × 10^4^ cells/well and incubated for 24 h. Cells were harvested by treating with Trypsin-EDTA (25200-072, Invitrogen). Cells were then stained by Trypan-Blue Solution (0.4%) (T8154, Sigma) and counted using a hematocytometer.

### Fluorescence microscopy

SHSY5Y or HeLa cells were seeded on 35 mm diameter confocal dishes (200350, SPL). For detection of mitochondrial morphology and mitochondrial membrane potential, cells were labelled with 0.2 μM Mitotracker Red CMXRos (M7512, Molecular Probes) or 250 nM Tetramethylrhodamine, Methyl Ester, Perchlorate (TMRM, T668, Molecular Probes) in DMEM medium for 30 min. After incubation, the fluorescent probe was washed out with PBS and confocal fluorescence images were obtained using an Olympus FV1200-OSR microscope. For detection of mitochondrial cytochrome c and mtDNA, cells were labelled with 0.2 μM Mitotracker Red CMXRos (M7512, Molecular Probes) for 30 min and fixed for 15 min at room temperature with 4% paraformaldehyde. Cells were permeabilized with 0.2% Triton X-100 for 10 min at room temperature and then incubated in blocking buffer (0.1% Triton X-100, 3% goat serum in PBS) for 40 min. Cells were incubated with an anti-DNA antibody (61014, PROGEN Biotechnik GmbH) or anti-cytochrome *c* antibody (556432, BD Pharmingen^™^) for overnight followed by incubation with anti-mouse Alexa-Fluor-488- conjugated secondary antibodies (A-11001, Life Technologies) for 1 h. Cells were washed three times for 5 min each with 1% Triton X-100 in PBS. During the final wash step, cells were incubated with 10 μg/ml DAPI (D1306, Life Technologies) in PBS for 5 min and analyzed for mtDNA and cytochrome c by fluorescence microscopy using an Olympus FV1200-OSR microscope. Image analysis was performed using Image J software. The percentage of mtDNA stain remaining was calculated using the following formula: (cDNA_v_ - nDNA_v_)/*n*, in which cDNA_v_ was the total cellular DNA volume determined by staining using anti-DNA antibodies, and nDNA_v_ was the total nuclear DNA stain volume determined using DAPI, where *n* denotes the number of cells.

Active caspase-3 was detected using the Image-iT LIVE Red Caspase Detection Kit (I35102, Life Technologies) according to the manufacturer's instructions. Briefly, cells were incubated with fluorescent inhibitor of caspases (FLICA) reagent for 60 min while protected from light. The solutions were then removed. After incubating with 1μM Hoechst for 2–10 min, confocal fluorescence images were obtained using an Olympus FV1200-OSR microscope.

### Luminescent assays

Cellular ATP levels were measured using CellTiter-Glo^®^ Luminescent cell viability assay kit (G7570, Promega) according to the manufacturer's instructions. Briefly, SHSY5Y or HeLa cells were plated on 96-well white-walled plates with clear bottoms in 100 μl culture media then 100 μl of CellTiter-Glo^®^ reagent was added to each well. The contents were mixed for 2 min on an orbital shaker to induce cellular lysis followed by incubation at room temperature for 10 min to stabilize the signal, then luminescence was recorded immediately.

Cellular ROS levels were measured using the ROS-Glo^™^ H_2_O_2_ Assay Kit (G8820, Promega) according to the manufacturer's instructions. Briefly, SHSY5Y or HeLa cells were plated on 96-well white-walled plates with clear bottoms in 80 μl culture media. To record luminescence, 20 μl H_2_O_2_ substrate solution was added to each well, and cells were incubated in a 37°C CO_2_ incubator for 2 hr. ROS-Glo^™^ detection solution (100 μl) was added to each well, and cells were incubated for 20 min at room temperature.

### Electron microscopy

Cells were fixed in 4% paraformaldehyde and 2.5% glutaraldehyde in a 0.1M phosphate buffer for overnight. After washing in a 0.1M phosphate buffer, cells were post-fixed with 1% osmium tetroxide in the same buffer for 1 h. Then, the samples were dehydrated with a series of the graded ethyl alcohol. The samples were embedded in Epon 812 and then polymerization was performed at 60°C for 3 days. Ultrathin sections (60–70 nm) were obtained by ultramicrotome (Leica Ultracut UCT). Ultrathin sections collected on grids (200 mesh) were examined in the transmission electron microscope (TEM) (JEOL JEM-1010) operating at 60 kV and images in the TEM were recorded by the CCD camera (SC1000; Gatan).

### Detection of cytosolic cytochrome c

SHSY5Y or HeLa cells were seeded in 100 mm diameter dishes (Thermo Scientific) and then transfected as indicated in the figure legends. After transfection, cells were harvested using a Qproteome Mitochondria isolation kit (37612, Qiagen) according to the manufacturer's instructions. Briefly, cells were lysed by adding Mitochondria Isolation Reagents A, B, and C, and the solution was centrifuged at 12,000 × g for 15 min to separate the cytosol and mitochondria fractions. Cytosol was used for western blot analysis.

### Measurement of oxygen consumption

Oxygen consumption measurements from cells were made using an XF96 Extracellular Flux Analyzer (Seahorse Bioscience). Briefly, cells were seeded at a density of 1.5 × 10^4^ cells per well in a XF96 cell culture microplate and incubated for 24 h to ensure attachment. Before assaying, cells were equilibrated for 1 h in unbuffered XF assay medium supplemented with 25 mM glucose, 1 mM sodium pyruvate, 2 mM glutamax, 1 × nonessential amino acids, and 1% FBS in a non-CO_2_ incubator. Mitochondrial processes were examined through sequential injections of oligomycin (1 μM), carbonyl cyanide 4-(trifluoromethoxy) phenylhydrazone (FCCP, 1 μM), and rotenone (1 μM)/antimycin A (1 μM). Indices of mitochondrial function were calculated as basal respiration rate (baseline OCR - rotenone/antimycin A OCR), ATP production (basal respiration rate - oligomycin OCR), and proton leak (oligomycin OCR - rotenone/antimycin A OCR).

### Statistical analysis

For statistical comparisons, *p* values were determined using Student's *t*-test or one-way ANOVA. A *p* value of < 0.05 was consider significant.

## SUPPLEMENTARY FIGURES


