# Supplementary material for: Tristetraprolin inhibits mitochondrial function through suppression of α-Synuclein expression in cancer cells
Source: Oncotarget. 2017 Mar 30;8(26):41903–20. doi: 10.18632/oncotarget.16706 (PMC5522037; doi:10.18632/oncotarget.16706)
Supplement: Supplementary file 1 [file oncotarget-08-41903-s001.pdf]

Tristetraprolin inhibits mitochondrial function through suppression of α-Synuclein expression in cancer cells

Supplementary Materials

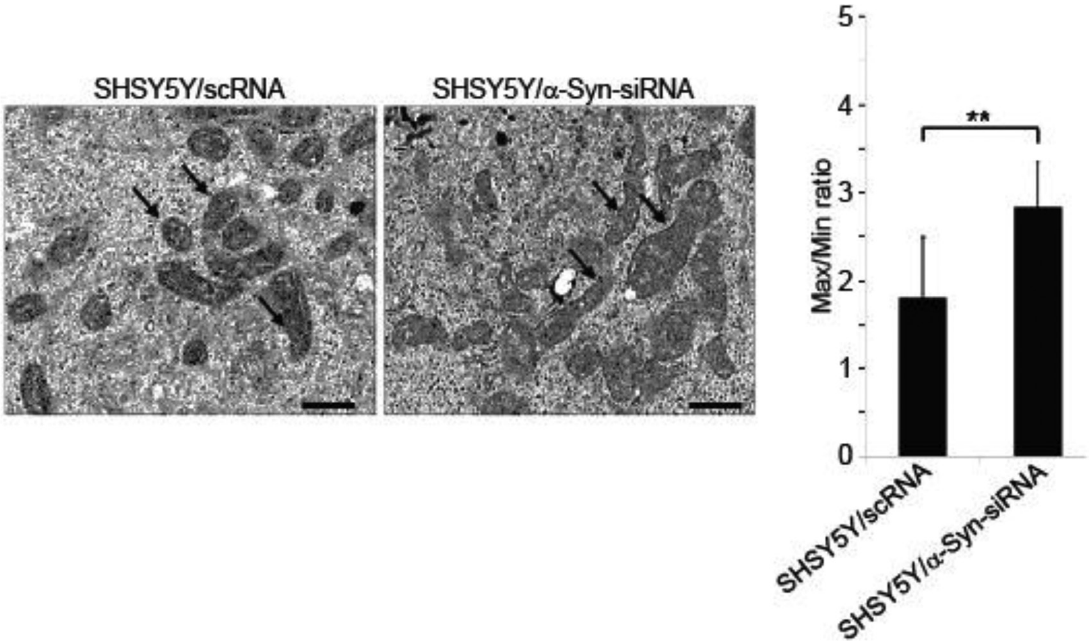

Supplementary Figure 1: Inhibition of α-Syn induces mitochondrial elongation. SHSY5Y cells were transiently transfected with scRNA (SHSY5Y/scRNA) or α-Syn-siRNA (SHSY5Y/α-Syn-siRNA) for 48 h. Representative electron microscopic images of mitochondria are displayed. Scale bar, 1 µm. Graphs represent ratio of maximum axis to minimum axis of mitochondria. Values are mean ± s.e.m. from three separate experiments (***p* < 0.01).

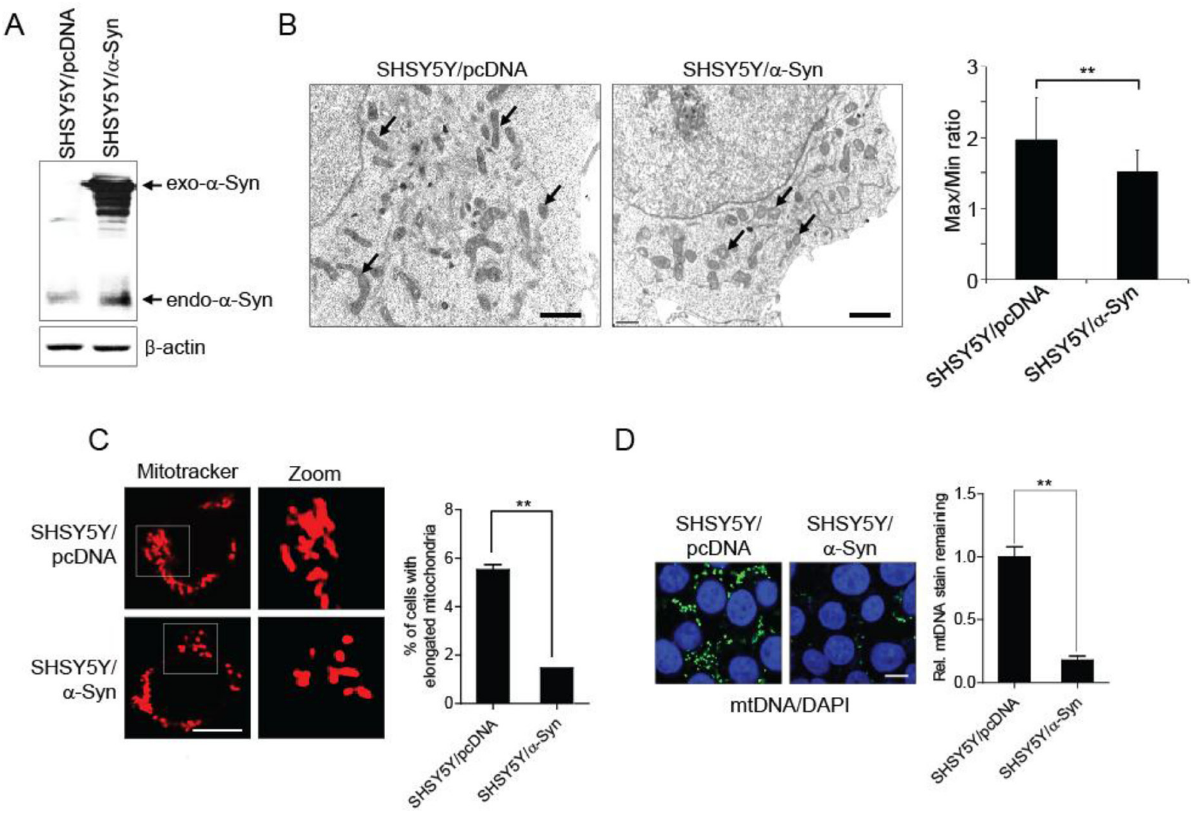

Supplementary Figure 2: Overexpression of α-Syn induces mitochondrial fragmentation. SHSY5Y cells were transiently transfected with pcDNA (SHSY5Y/pcDNA) or pcDNA/α-Syn (SHSY5Y/α-Syn) for 48 h. (**A**) α-Syn levels were determined by western blot. (**B**) Representative electron microscopic images of mitochondria. Scale bar, 1 μm. Graphs represent ratio of maximum axis to minimum axis of mitochondria. Values are mean ± s.e.m. from three separate experiments (**$p < 0.01$). (**C**) Cells were stained with Mitotracker CMXRos for 30 min, and images were obtained by confocal microscopy. The panels display representative confocal images with magnified insets of boxed areas. Graphs represent percentage of cells with elongated mitochondria. Values are mean ± s.e.m. from three separate experiments with 100 cells per group per experiment (**$p < 0.01$). (**D**) Cells were stained with anti-DNA antibody for mtDNA (green) and DAPI for nuclear DNA (blue). The panels display representative confocal images. Scale bar, 10 μm. Graphs represent relative percentage of mtDNA stain remaining. Values are mean ± s.e.m. from three separate experiments with 100 cells per group per experiment (**$p < 0.01$).
